# Perceived workplace and private life discrimination during legally mandated COVID-19 quarantine: Evidence from a large municipal cohort in Cologne, Germany

**DOI:** 10.3205/dgkh000673

**Published:** 2026-09-07

**Authors:** Sisai Sadiq, Sven Feddern, Anna Carlotta Graf, Barbara Grüne, Luis Haberstock, Annelene Kossow, Johannes Niessen, Susanne Rost, Nikola Schmidt, Gerhard A. Wiesmüller, Christine Joisten

**Affiliations:** 1 Department for Physical Activity in Public Health, German Sports University, Institute of Movement and Neurosciences, Cologne, Germany; 2 Institute for Occupational, Social and Environmental Medicine, Medical Faculty, RWTH Aachen University, Aachen, Germany; 3 Formerly Cologne Health Department, Infektions- und Umwelthygiene, Cologne, Germany; 4 Cologne Health Department, Infektions- und Umwelthygiene, Cologne, Germany; 5 Augsburg District Health Department, Augsburg, Germany; 6 Institute of Hygiene, University Hospital Muenster, Münster, Germany

**Keywords:** COVID-19, quarantine, perceived discrimination, social stigma, psychosocial stress, public health, social exclusion

## Abstract

**Aim:**

During the COVID-19 pandemic, quarantine measures were widely implemented and may have contributed to perceived discrimination. However, empirical evidence on its prevalence and associated factors remains limited. This study aimed to assess perceived discrimination in workplace and private contexts during quarantine and to identify associated factors.

**Methods:**

Data from 3,853 participants of the CoCo-Fact cohort study (Cologne Corona counselling and support for index and contact persons during the quarantine period) in Cologne, Germany were analysed. Perceived discrimination was assessed using a single-item measure (yes/partly/no), complemented by qualitative free-text responses. Logistic regression models were used to identify associated factors.

**Results:**

Perceived discrimination was reported by 5.3% of participants in the workplace and by 6.9% in private life. Workplace discrimination was primarily organisational or economic, while private-life discrimination was predominantly psychosocial. Associated factors included migrant background, higher psychological distress, and lower coping skills. Additional factors associated with private-life discrimination included younger age, higher socioeconomic status, and unemployment. The model explained 8.3% and 14.2% of the variance, respectively.

**Conclusion:**

Although overall prevalence was low, vulnerable groups were disproportionately affected. Findings highlight the need for targeted psychosocial and structural support measures in future public health crises.

## Zusammenfassung

**Ziel:**

Während der COVID-19-Pandemie wurden umfangreiche Quarantänemaßnahmen umgesetzt, die möglicherweise zu Diskriminierungserfahrungen beigetragen haben. Empirische Erkenntnisse zur Prävalenz und zu assoziierten Faktoren solcher Erfahrungen sind bislang begrenzt. Ziel dieser Studie war es, Diskriminierungserfahrungen im Arbeits – und Privatleben während der Quarantäne zu untersuchen und damit assoziierte Faktoren zu identifizieren.

**Methoden:**

Analysiert wurden Daten von 3.853 Teilnehmenden der CoCoFakt-Kohorten-Studie (Cologne-Corona-Beratung und Unterstützung für Index- und Kontakt-Personen während der Quarantäne-Zeit) in Köln. Diskriminierungserfahrungen wurden mittels einer Single-Item-Frage (ja/teilweise/nein) erfasst und durch qualitative Freitextantworten ergänzt. Zur Identifikation assoziierter Faktoren wurden logistische Regressionsmodelle verwendet.

**Ergebnisse:**

Diskriminierungserfahrungen wurden von 5,3% der Teilnehmenden am Arbeitsplatz und von 6,9% im Privatleben berichtet. Diskriminierung am Arbeitsplatz war vorwiegend organisatorisch/wirtschaftlicher Natur, während Diskriminierung im Privatleben vor allem psychosoziale Aspekte betraf. Zu den assoziierten Faktoren zählten Migrationshintergrund, höhere psychische Belastung und geringere Bewältigungskompetenzen. Weitere mit Diskriminierung im Privatleben assoziierte Faktoren waren jüngeres Alter, höherer sozioökonomischer Status und Arbeitslosigkeit. Die Modelle erklärten 8,3% bzw. 14,2% der Varianz.

**Schlussfolgerungen:**

Obwohl die Gesamtprävalenz gering war, waren vulnerable Gruppen überproportional betroffen. Die Ergebnisse unterstreichen die Notwendigkeit gezielter psychosozialer und struktureller Unterstützungsmaßnahmen in zukünftigen Public-Health-Krisen.

## Introduction

In December 2019, the first outbreaks of COVID-19 caused by SARS-CoV-2 were first reported in Wuhan, China [1]. To contain its spread, governments worldwide imposed restrictions ranging from social distancing and contact restrictions to quarantine and home isolation for infected persons (IPs) and contact persons (CPs) [2]. In Germany, quarantine or home isolation following a diagnosis or contact with a confirmed COVID-19 case lasted 10–14 days during the study period, based on the applicable coronavirus protection regulations. Possible consequences of loneliness due to isolation measures included an increased tendency towards depression, suicidal thoughts and actions, and post-traumatic stress disorder [3], [4]. Discrimination also occurred due to stigmatisation in the context of COVID-19 infection, even without proven contact with the pathogen. In Italy, members of the Chinese community were ostracised even before the national lockdown: Chinese restaurants were deserted, and parents did not want to send their children to school because of their Chinese classmates [5]. Increased cases of xenophobia occurred towards people of Chinese descent, including violent riots [6]. Subsequently, infected individuals, close contacts, healthcare workers, and family members of COVID-19 patients were marginalised in many countries [7], [8]. In India, infections led to family rejection, neighbourhood marginalisation and social media harassment. For example, a pregnant woman who had contracted COVID-19 was rejected by her family after giving birth in hospital [7]. In another case, an entire street was declared a ‘corona road’ and shunned because a COVID-19 ‘survivor’ lived there [7]. 

Also outbreaks of other infectious diseases such as HIV, tuberculosis, leprosy and Ebola, have led to discrimination of those affected [9], [10]. During the 2002/2003 SARS-CoV-1 pandemic, Hawryluck et al. [11] conducted an online survey on the psychological effects of quarantine: 51% of the participants said that they had noticed a change in behaviour in their social environment – they felt shunned, received fewer phone calls and were no longer invited to events. In February 2020, the World Health Organization (WHO), United Nations Children’s Fund (UNICEF) and the International Federation of Red Cross and Red Crescent Societies (IFRC), published a guide on how to recognise social stigmatisation due to COVID-19 infections early and prevent consequences, warning that fear of possible discrimination could discourage testing or medical care and lead to a more rapid spread of the virus [10]. 

Consistent with stigma and discrimination research, perceived discrimination is understood as subjectively perceived unfair treatment or judgement with research noting wide variability in how discriminatory experiences are captured [12], [13]. It has been consistently associated with adverse mental and physical health outcomes, including increased stress responses and poorer psychological well-being, regardless of the sources of discrimination [13], [14]. By July 2020, the Federal Anti-Discrimination Agency in Germany had already recorded around 700 requests for advice on discrimination related to COVID-19 [15]. Experiences of discrimination are a recurring phenomenon during infectious disease outbreaks and are not limited to the acute phase of a pandemic [16]. Beyond crisis-specific circumstances, discrimination reflects enduring psychosocial and structural mechanisms related to stigma, social exclusion, and inequality. Legally mandated quarantine measures may act as a trigger that amplifies pre-existing vulnerabilities, leading to long-term consequences even after quarantine has ended [17]. 

This analysis draws on the Health Stigma and Discrimination Framework to situate perceived discrimination as an experienced manifestation of stigma shaped by individual and structural factors, with implications for mental health and social participation [18]. Understanding these mechanisms is essential for designing resilient public health responses in future pandemics and other public health emergencies. However, little data is currently available that can be used to develop specific recommendations and effective countermeasures in future pandemic scenarios. As part of the second wave of the CoCo-Fakt study conducted by one of Germany’s largest health authorities, we examined how infected individuals and their relevant contacts experienced discrimination as subjectively perceived unfair or exclusionary treatment following legally mandated quarantine and which factors were associated with their perception. 

In this study, perceived discrimination is operationalised as subjectively experienced exclusion or unfair treatment during quarantine, rather than legally defined discrimination.

## Materials and methods

### Study design

Since the beginning of the pandemic, laboratory-confirmed SARS-CoV-2 infections have been notifiable in Germany, with responsible laboratories reporting all positive findings to the respective health authorities. In Cologne, infected persons (IPs; reported as positive for the virus) were contacted by the Health Authority, placed under mandatory quarantine, and their demographic, socioeconomic, and health data and relevant contact persons (CPs) were entered into the DiKoMa system (digital contact management; a database developed by the City of Cologne’s Office for Information Processing [19]). CPs were defined as persons who had contact with a confirmed COVID-19 case during the infectious period.

The CoCo-Fakt study tracked three waves (Wave 1: 28 February to 9 December 2020; Wave 2: 1 January to 30 June 2021; Wave 3: 1 July to 31 December 2021) of individuals who were placed under home isolation or quarantine by the Cologne Health Authority due to either a positive COVID-19 test or relevant contact. Eligible participants were identified via DiKoma and invited by email to complete an online survey. Persons under the age of 16 years, those without a declaration of consent, non-compliant persons, deceased patients and patients in medical or nursing facilities were excluded. The survey was conducted using Unipark software. 

The mixed-methods online survey was based on the COVID-19 Snapshot Monitoring Study conducted by the University of Erfurt and the WHO [20]. The questionnaire was available in German, Turkish and English in the first wave, in German, Turkish, Arabic and Bulgarian in the second wave, and in German and Turkish in the third wave. Completion time was approximately 35 minutes. The comprehensive study design, including the original questionnaire, has already been published [21].

### Study population

The analysis included only participants from the second wave of the survey. Individuals who met the inclusion criteria (n=37,532) received an email with a link to the online survey. Reminders were sent approximately 2 and 4 weeks after the initial invitation. All individuals over the age of 16 with complete information on sex, quarantine reason and information on perceived discrimination due to quarantine were included in the final sample, summarised in Figure 1 (Fig. 1).

### Questionnaire

#### Demographic data

The reason for home isolation or quarantine (IP or CP), age, sex (male or female), and education (based on the highest school and professional qualification) were recorded. Educational levels were classified according to the German Health Interview and Examination Survey for Adults (DEGS1) [22] into ‘high’, ‘medium’, and ‘low’. For the analysis, the categories ‘medium’ and ‘low’ were combined due to their limited number. Current occupational status (worker, employee, civil servant, freelancer or other self-employed person, assisting family member) and employment status (full-time, part-time and occasional or irregular, not employed) were also collected. To indicate migration background, the main language spoken at home was recorded and classified as no (German) or yes (all other languages).

#### Health status

Height in cm and weight in kg were recorded and used to calculate the body mass index (BMI) and classified as follows: underweight (BMI <18.5 kg/m^2^), normal weight (BMI 18.5–24.9 kg/m^2^), overweight (BMI 25.0–29.9 kg/m^2^), and obese (BMI ≥30.0 kg/m^2^) [23]. In addition, participants stated whether chronic diseases were present [23].

#### Personal situation

Details were collected on living in a partnership (yes or no), the number of people over 16 years of age in their household, and the number of children under 16 years of age.

#### Living situation

We recorded whether the person lived alone in the household during isolation or quarantine (yes, no) and whether they had a balcony or garden (none, balcony, garden, both).

#### Mental health

Mental stress during home isolation and quarantine [24] was recorded using four items adapted from the COSMO study [20]

- I felt nervous, anxious or tense (item 1, GAD-7 [25])
- I felt down or depressed (item 6, ADS [26], [27])
- I felt lonely (item 14, ADS [26], [27])
- Thoughts about my experiences during the coronavirus pandemic triggered physical reactions such as sweating, shortness of breath, dizziness or palpitations (item 19, IES-R [28]).

Responses were rated on a 6-point Likert scale from ‘not at all/less than 1 day’ to ‘always/daily’ and summarised into four categories (‘not at all’, ‘1 to 2 days’, ‘3 to 4 days’ and ‘5 to 7 days’). The psychological distress score was calculated as the mean of the individual items, with lower score indicating less distress. The internal consistency of the score was good (Cronbach’s alpha = .805; subscale reliability = .468 to .765). Suicidal tendencies were not recorded.

#### Coping and support

The use of possible support systems, such as help from neighbours or friends, was recorded and rated from ‘does not apply at all’ (1) to ‘applies completely’ (6), as well as personal assessments (e.g. ‘I had a plan for my daily routine in terms of sleep, work or physical activities’, ‘I communicated with family, friends and acquaintances via digital media’) and open-ended questions. The coping score was calculated by the mean of the individual items, with higher scores indicating better coping skills and abilities [24]. Internal consistency was acceptable (Cronbach’s alpha = .702; subscale reliability .305 to .571).

#### Assessment of discrimination during the quarantine period

Participants were asked whether they experienced exclusion in their workplace or private environment during quarantine (‘yes’, ‘partly’, ‘no’). In this study, perceived discrimination was operationalised as subjectively perceived exclusion or unfair treatment.

The assessment relied on a single-item measure. In addition, qualitative free-text responses were collected and analysed using structured content analysis, considering perceived discrimination in the workplace and in private life separately [29]. Categories were defined inductively based on a scientific literature review [30], [31] and included

1. ‘discrimination at an organisational or economic level’ (e.g. ‘I was unable to go on a business trip’),
2. ‘discrimination at a psychosocial level’ (e.g. ‘My flatmate didn’t talk to me for weeks’) and
3. ‘Other’.

The categorisation is shown in Table 1 (Tab. 1).

### Data evaluation and analyses

SPSS (Version 29.0) was used to perform all data analyses, and MAXQDA was used to analyse the qualitative data. Associations between participant characteristics (e.g. age, sex and SES) were examined using Χ^2^ tests, independent t-tests or analysis of variance. The effect sizes were calculated using Cramer’s V (Χ^2^ tests; small: 0.06–0.15; medium: 0.16–0.26; large: ≥0.26) or Cohen’s d (independent t-test; trivial: <0.2; small: 0.2–0.5; medium: 0.5–0.8; large: ≥0.8). The internal consistency of the scores was determined using Cronbach’s alpha. Values ≥0.8 were considered good internal consistency, and values ≥0.7 were considered acceptable. 

Binary logistic regression models were calculated to examine possible associated factors for perceived discrimination in the workplace or in private life. For this purpose, responses ‘yes’ and ‘partly’ were combined and compared with the response option ‘no’. The significance, odds ratios (OR) and 95% confidence intervals (CI) were determined for the following factors associated with: age (in years), psychological distress score (metric), coping score (metric), reason for quarantine (IP=0; CP=1), sex (female=1, male=2), migration background (no=0, yes=1), SES (low or medium=0, high=1), employment (not or irregular employed=0; part- or full-time=1), and living situation (no garden/no balcony=0; garden or balcony or both=1). Nagelkerke’s pseudo R^2^ was used to assess model fit. The significance level was set α=.05. The relatively low explained variance indicates that additional unmeasured psychosocial and contextual factors may contribute to perceived discrimination.

### Ethical approval

The study was approved by Rheinisch-Westfälische Technische Hochschule (RWTH) Aachen Human Ethics Research Committee (351/20).

## Results

### Study population and demographic data

After cleaning the database of non-responders, 6,965 records remained. A total of 3,853 participants were included, comprising 2,199 IPs (57.1%) and 1,654 CPs (42.9%). The average age of the entire sample was 43.0 years (SD=14.4), and 60.6% were female. The proportion of women was significantly lower among IPs (59.0%) than among CPs (62.7%; p=.021). Overall, 9.2% of participants reported a migrant background (11.4% of IPs vs. 6.3% of CPs; p<.001). 

Employment status differed significantly between groups: IPs were less likely to be in full- (58.7% vs. 60.0%) or part-time employment (21.7% vs. 23.5%) and more often not employed (16.2% vs. 13.0%; p=.041) compared to CPs. IPs also reported significantly more often than CPs that they had children under the age of 16 years (25.5% vs. 22.6%; p=.035). Regarding the living situation, 23.5% had a garden (22.2% vs. 25.3%), and 50.6% a balcony (52.7% vs. 47.8%; p=.014). Other parameters showed no significant differences between groups. Participants characteristics are summarised in Table 2 (Tab. 2). 

### Sum scores for psychological stress and coping skills

The mean psychological distress score across all participants was 2.5 (SD=1.3). Among IPs, the mean score averaged 2.7 (SD=1.4), which was significantly higher than among CPs, where it averaged 2.4 (SD=1.3; *p*<.001) (Table 3 (Tab. 3)).

The overall mean coping competence score was 4.3 (SD=1.0). For CPs, it was significantly higher at 4.4 (SD=1.0) than among IPs (4.3; SD=1.0; *p*<.001) (Table 3 (Tab. 3)).

### Information on perceived discrimination

Of the 3,853 participants, 102 (2.6%) perceived discrimination in the workplace. A total of 104 people (2.7%) answered the question with ‘partly’, while 3,747 (94.7%) reported no perceived discrimination. No significant differences were found between IPs and CPs (*p*=.394; Table 4 (Tab. 4)).

Regarding perceived discrimination in private life, 116 participants (3.0%) answered ‘yes’ and 151 (3.9%) ‘partly’, whereas 3,586 people (93.1%) reported no perceived discrimination. Again, no differences were found between groups (*p*=.388; Table 4 (Tab. 4)). 

### Analyses of free-text responses

In total, 313 qualitative responses were analysed (192 IP; 121 CP). Among IPs, 51.8% described perceived discrimination in the workplace at the organisational or economic level, and 34.1% reported discrimination at the psychosocial level; 14.1% of the responses fell into the ‘other’ category. In the CP group, perceived discrimination was also predominantly reported at the organisational or economic level (65.5%); 27.6% perceived discrimination at the psychosocial level, and 6.9% of responses fell into the ‘other’ category (6.9%) (Figure 2 (Fig. 2)).

In their private lives, 59.2% of IPs reported perceived discrimination at the psychosocial level and 30.0% at the organisational or economic level, and 10.8% of the responses were assigned to the ‘other’ category. In the CP group, 39.7% perceived discrimination in private life at the organisational or economic level and 46.6% at the psychosocial level, with 13.7% of the responses being classified as ‘other’. The relative distribution of the categories is shown in Figure 3 (Fig. 3).

### Regression analyses

The probability of perceived discrimination in the workplace (‘yes’ or ‘partly’) was increased by 85% among participants with a migrant background (OR: 1.85; 95% CI: 1.17–2.93), by 41% with higher psychological distress (OR: 1.41; 95% CI: 1.23–1.61) and lower coping skills by 26% (OR: 0.79; 95% CI: 0.66–0.95) (Table 5 (Tab. 5)). No influence was found in terms of sex, age, SES, employment type or quarantine reason (IP or CP). The model explained 8.3% of the variance.

The probability of perceived discrimination in private life was increased by 1% for younger age (OR: 0.99; 95% CI: 0.97–1.00), by 66% among participants with migrant background (OR: 1.66; 95% CI: 1.09–2.51), by 49% for those with high SES (OR: 1.49; 95% CI: 1.02–2.16), by 33% for unemployment (OR:1.33; 95% CI: 1.16–1.51), by 49% for higher psychological distress (OR: 1.49; 95% CI: 1.32–1.68) and by 36% for lower coping skills (OR: 0.73; 95% CI: 0.62–0.85) (Table 6 (Tab. 6)). No influence was found in terms of sex or quarantine reason (IP or CP). The model explained 14.2% of the variance.

Overall, the regression models explained a limited proportion of variance, suggesting that additional factors beyond those included in the models influence perceived discrimination.

## Discussion

The study aimed to examine the effects of legally mandated quarantine measures during the COVID-19 pandemic on perceived discrimination in both workplace and private contexts, and to identify possible associated factors. Overall, 5.3% of respondents reported perceived discrimination in the workplace, and 6.9% reported perceived discrimination in their private lives. In the workplace, perceived discrimination mainly occurred at the organisational or economic level (57.3%). Perceived discrimination in the workplace was associated with a migration background (85%), higher psychological distress (41%) and lower coping skills (26%). 

In the private sphere, perceived discrimination at the psychosocial level tended to be more prominent (54.4%). Perceived discrimination was associated with young age (1%), migrant background (66%), high SES (49%), unemployment (33%), higher psychological distress (49%), and lower coping skills (36%).

Although overall prevalence was low, vulnerable groups were affected more frequently. Similarly, Devakumar et al. [32] observed that individuals with a migrant background suffered disproportionately high from the pandemic due to structural disadvantages, including limited options for self-isolation and fear of discrimination, which sometimes led to concealment of symptoms. In Germany, this group experienced higher infection rates [33], with household size, language barriers and low SES also being additional factors. These structural disadvantages not only contributed to an increased infection rate and more frequent quarantine requirements but also reinforced experiences of social discrimination – a self-reinforcing vicious circle that represents a further dimension of disadvantage. Interestingly, in our study, a high SES also emerged as a risk factor, possibly due to more visible social roles, larger social networks, and greater awareness of subtle discrimination, increasing both exposure and reporting. 

Analyses of the Mannheim Corona Study at the beginning of the pandemic showed that the proportion of unemployed people in lower education and income groups was significantly higher than in other segments of the population. Low-income individuals, in particular, were more affected by a lack of short-term work and higher unemployment at the beginning of the pandemic [34]. Precarious financial situations increase both socioeconomic burden and the risk of experiencing discrimination. Financial and job losses during the COVID-19 pandemic were additionally associated with adverse psychological effects [35], further increasing the risk of discrimination.

The results of this study are consistent with findings from previous epidemics. Government-imposed quarantine measures during the 2014 Ebola epidemic in Liberia led to increased condemnation, stigmatisation and serious socioeconomic burdens for those affected [36]. Similarly, during the 2015 Middle East respiratory syndrome epidemic in Korea, affected individuals experienced prolonged psychological symptoms such as anxiety and anger, exacerbated by financial losses and pre-existing psychological stress [37]. The meta-analysis by Neelam et al. [38] showed that people with pre-existing mental illnesses were at greater risk of developing further and more severe psychological symptoms during pandemics and had higher psychiatric morbidity than the healthy control group. Our findings support this insight by showing that psychological distress was a decisive factor in the subjective perception of workplace and private discrimination during the COVID-19 pandemic. While isolation and quarantine measures remain essential to control the spread of infectious diseases and thus an epidemic or pandemic, these examples show that can long-term negative consequences also occur. 

To address the resulting challenges, low-threshold support services must be developed, especially in the context of psychosocial support. Telephone counselling services available in Germany, such as the telephone counselling hotline (TelefonSeelsorge), the youth helpline (Nummer gegen Kummer), the violence against woman helpline (Hilfetelefon Gewalt gegen Frauen), the parental counselling helpline (Elterntelefon), and the German Depression support hotline (Deutsche Depressionshilfe) should be easily accessible, multilingual, free of charge and, if necessary, anonymous. Short-term support initiatives, such as the Corona Hotline by the Association of German Psychologists (Berufsverband Deutscher Psychologinnen und Psychologen e.V.) provided free and anonymous support but were limited to the German-speaking population and eventually discontinued, as they were run by volunteer therapists, which proved unsustainable during the prolonged pandemic period. Additionally, digital support services such as Psychological Corona Help (Psychologische Coronahilfe) were launched, but these too were accessible only to the German-speaking population with internet access. Financial aid [39] also provided relief, but the bureaucratic hurdles were very high and often linguistically challenging. Consequently, access to social, financial, and health support services must be simplified and made available in multiple languages. Moreover, such services should be expanded, institutionalised, and widely promoted as part of crisis preparedness strategies, ensuring availability and visibility in future public health emergencies such as pandemics. 

Regarding discrimination, stigmatisation and other forms of marginalisation in the context of the coronavirus pandemic, basic monitoring and research programmes already exist. These include the StiPex project (Stigmatisation in the context of the coronavirus pandemic: exploration of psychosocial processes and intersectional aspects and their significance for prevention) at the University of Greifswald, which used population surveys, among other instruments, to record health-related stigmatisation during COVID-19 in Germany. Its goal is to analyse and process stigmatisation processes and existing anti-stigma practices to detect reinforcing factors, the manifestation of stigmatisation, its behavioural and health-related consequences and social impacts, to develop suitable measurement instruments and formulate recommendations for action [40].

The framework model is based on the Health Stigma and Discrimination Framework by Stangl et al. [18], which roughly describes stigmatisation processes as a sequence of drivers, experiences and outcomes. Migration background and SES act as drivers, discrimination as experience, and psychological stress may act as both a driver (increased vulnerability) and an outcome. This theoretical foundation provides a crucial basis for developing targeted measures that foster solidarity and resilience among the population. Translating these principals into practice requires strengthening community-based structures, including neighbourhood support, volunteer engagement, and self-help initiatives, which offer trust-based assistance that formal systems alone cannot provide. Public institutions should coordinate, facilitate and promote these networks to enhance their visibility and effectiveness. 

## Strengths and limitations

A key strength of this study is the large dataset collected by the Cologne Health Authority and the fact that only legally quarantined persons were included. The evaluation of free-text responses allowed for individual perspectives and subjective perceptions of discrimination to be recorded. Nevertheless, several limitations must be noted. Firstly, selection bias must be considered. Individuals with higher SES and better access to digital resources were overrepresented, whereas vulnerable populations may be underrepresented. This may have led to an underestimation of discrimination prevalence, particularly among socially disadvantaged or harder-to-reach groups. Secondly, some of the interviews were conducted several months after quarantine, so recall bias cannot be ruled out. It is unclear whether respondents referred exclusively to their quarantine experiences or also to later pandemic experiences. Thirdly, a key methodological consideration is the measurement of perceived discrimination. The use of a single-item measure allowed for pragmatic data collection in a large population-based survey but limits construct validity and does not allow differentiation between frequency, severity, or specific types and dimensions of discriminatory experiences. While validated multi-item instruments such as the Everyday Discrimination Scale exist [41] , their use was not feasible within the survey design. The findings should be interpreted in light of the distinction between perceived discrimination and objectively measurable discrimination, as subjective perceptions may be influenced by psychological distress or coping capacity. The concept assessed in this study may therefore more closely reflect perceived social exclusion or unfair treatment rather than legally defined discrimination. However, the inclusion of qualitative data provides important contextual insights and supports interpretation of the findings. 

The relatively low explained variance of the regression models indicates that perceived discrimination is influenced by additional psychosocial, contextual, and structural factors, that were not captured in this study. 

Additionally, one item (“I felt hopeful about the future”, item 8, ADS [25], [26]), originally part of the adapted COSMO-based mental stress subscale, was excluded due to poor internal consistency. The Cronbach’s alpha of the original five-item scale was very low (α=.084), whereas exclusion of the item improved reliability to an acceptable level (α=.805). 

Furthermore, migration background was operationalised using language spoken at home, which does not capture visible markers of ethnicity or experiences related to racialisation. In addition, generalisability is also limited as the cohort is municipal and data were collected as part of the second wave of the CoCo-Fakt study (1 January to 30 June 2021). The results must be interpreted in the context of the early national COVID-19 vaccination campaign, the quarantine regulations in force at the time, and the protective measures introduced in April 2021 (‘federal emergency brake’). 

Due to the low subscale reliability of the item ‘I felt hopeful about the future’ (Cronbach’s α=.084), it was removed from the scale.

## Conclusion

Although only a small proportion of participants reported perceived discrimination, vulnerable groups were disproportionately affected. These findings highlight the importance of addressing psychosocial and structural inequalities in public health responses. Future pandemic preparedness strategies should include accessible, multilingual, and low-threshold psychosocial support services, targeted anti-stigma communication, and community-based support structures to strengthen social resilience during public health crises

## Notes

### Authors’ ORCIDs

- Sadiq S: https://orcid.org/0009-0006-0263-4383
- Grüne B: https://orcid.org/0000-0003-4393-333X
- Kossow A: https://orcid.org/0000-0002-4648-4851
- Schmidt N: https://orcid.org/0000-0001-8739-8354
- Wiesmüller GA: https://orcid.org/0000-0001-5478-171X
- Joisten C: https://orcid.org/ 0000-0002-2455-8901

### Ethical approval 

The study was approved by Rheinisch-Westfälische Technische Hochschule (RWTH) Aachen Human Ethics Research Committee.

### Funding

None.

### Availability of data and materials

The data sets used and/or analysed in the present study are not publicly available due to the inclusion of sensitive personal data.

### Generative AI Statement 

The authors declare that generative AI (ChatGPT, OpenAI; GPT-4) was used exclusively to assist with language editing, grammatical refinement, and improvement of clarity of expression. The authors reviewed and edited the manuscript and take full responsibility for its content.

### Competing interests

The authors declare that they have no competing interests.

## Figures and Tables

**Table 1:**
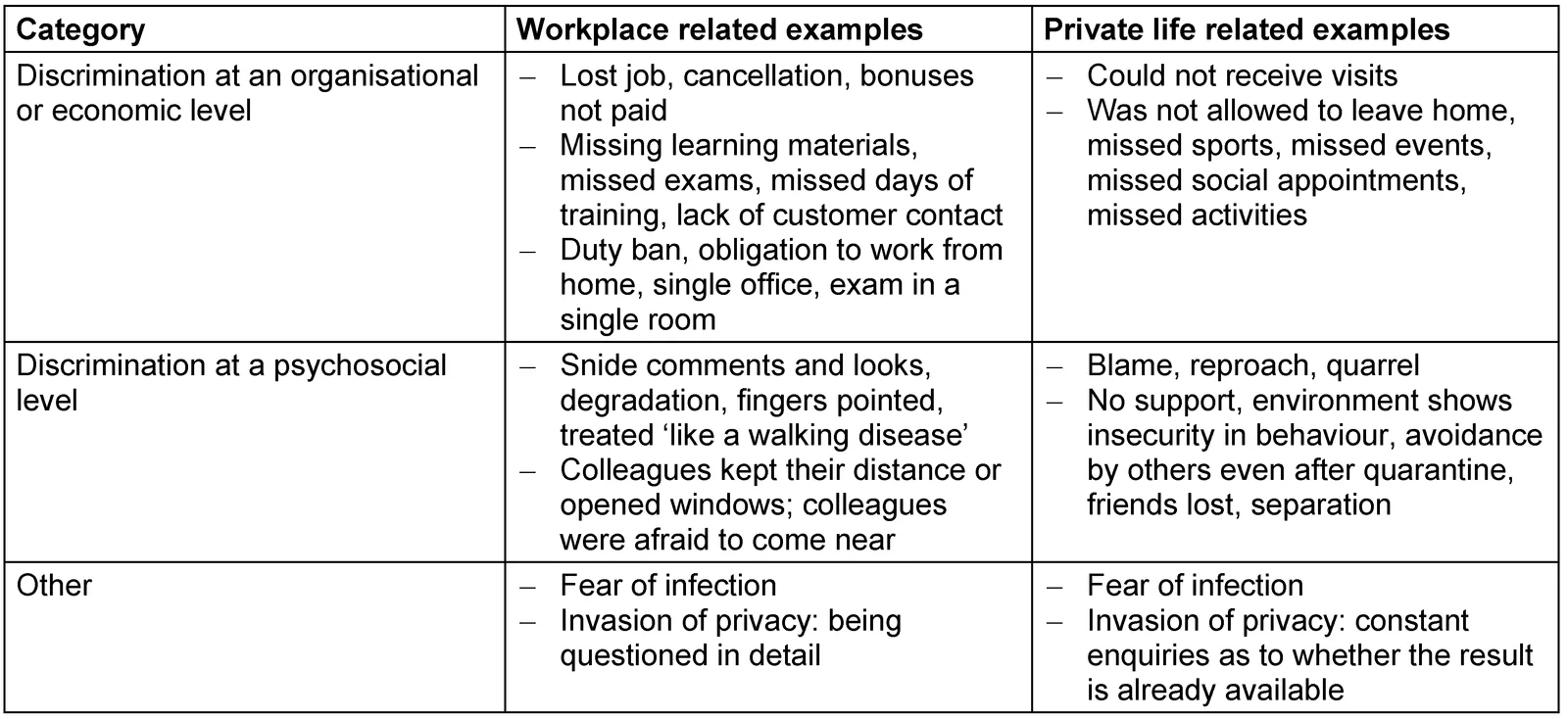
Categories of perceived discrimination with illustrative examples from qualitative responses

**Table 2:**
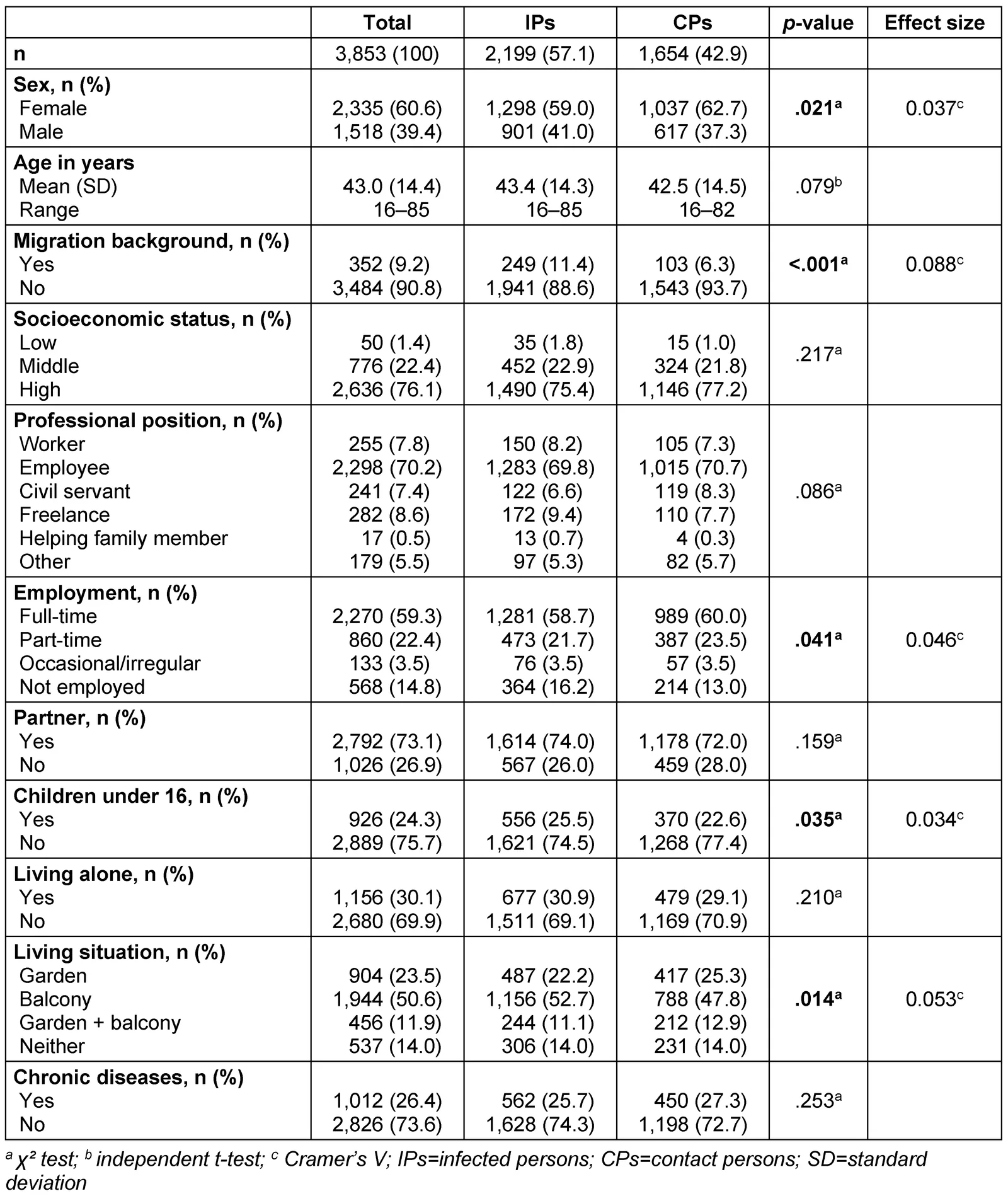
Demographic characteristics

**Table 3:**
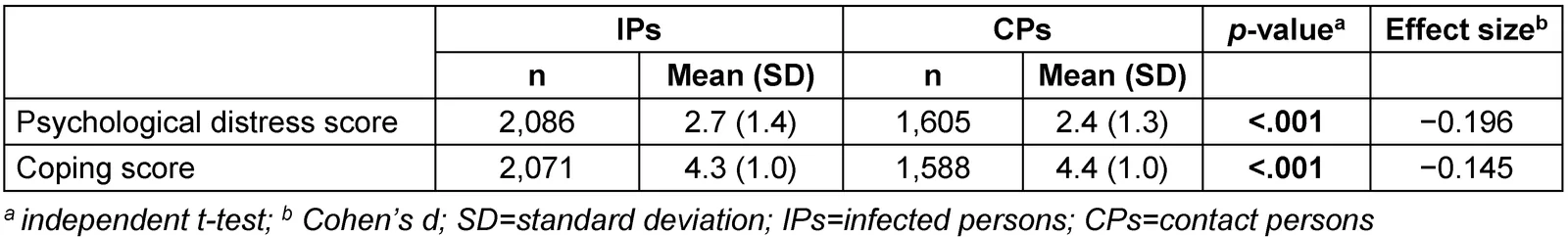
Sum scores of coping and psychological distress

**Table 4:**
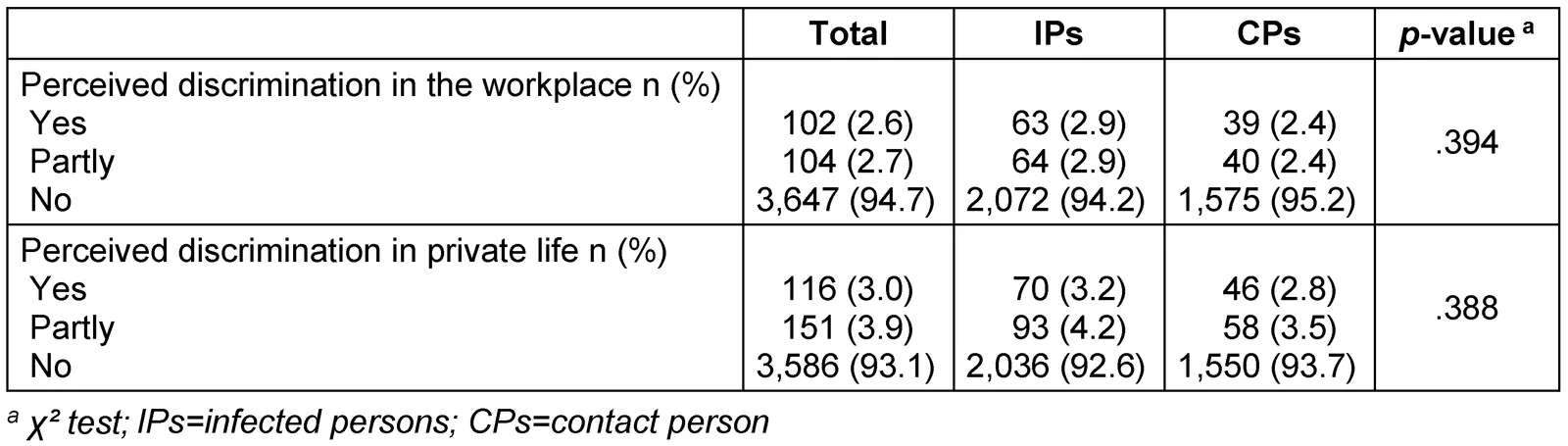
Perceived discrimination in the workplace and in private life

**Table 5:**
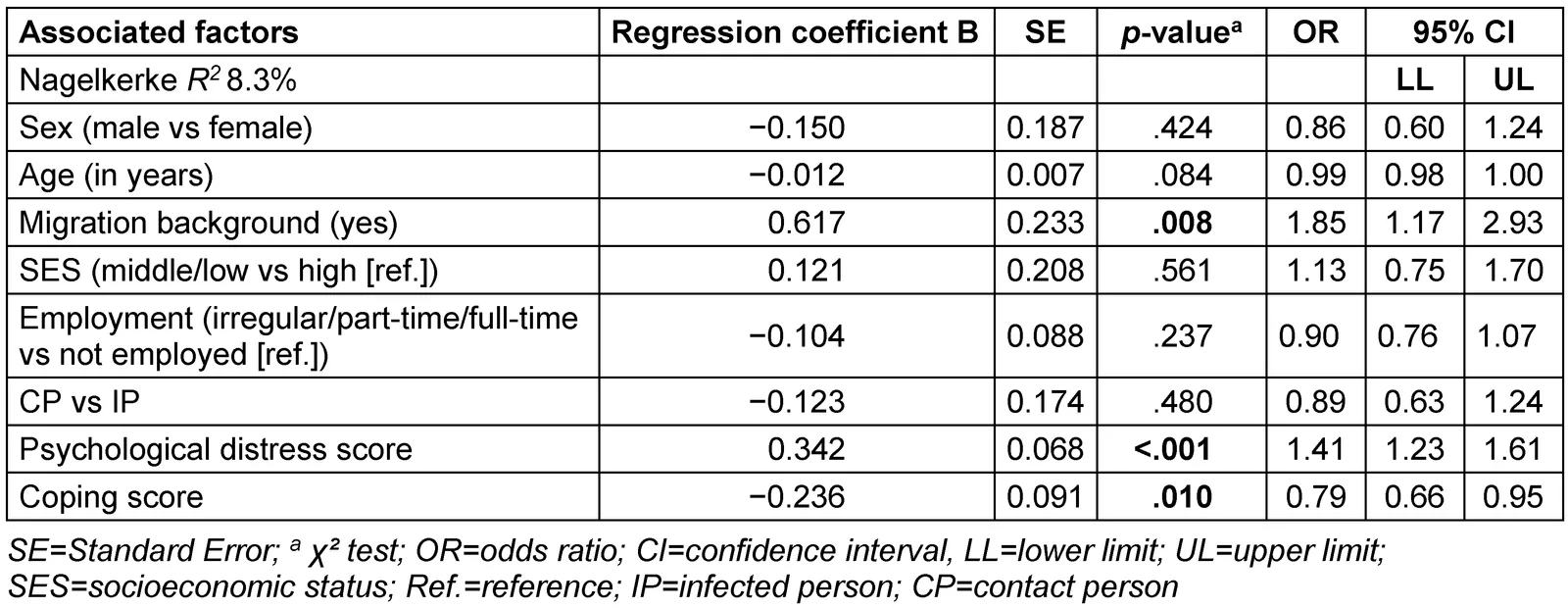
Factors associated with perceived discrimination in the workplace (logistic regression analysis)

**Table 6:**
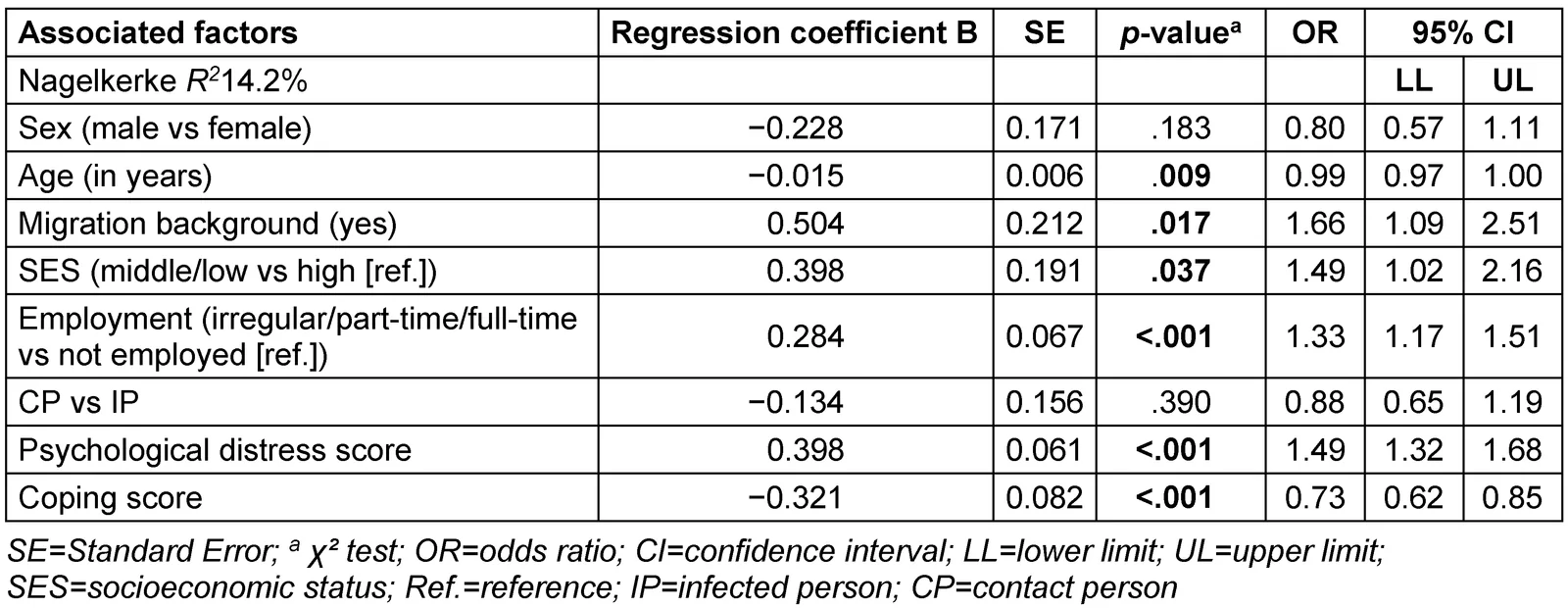
Factors associated with perceived discrimination in private life (logistic regression analysis)

**Figure 1:**
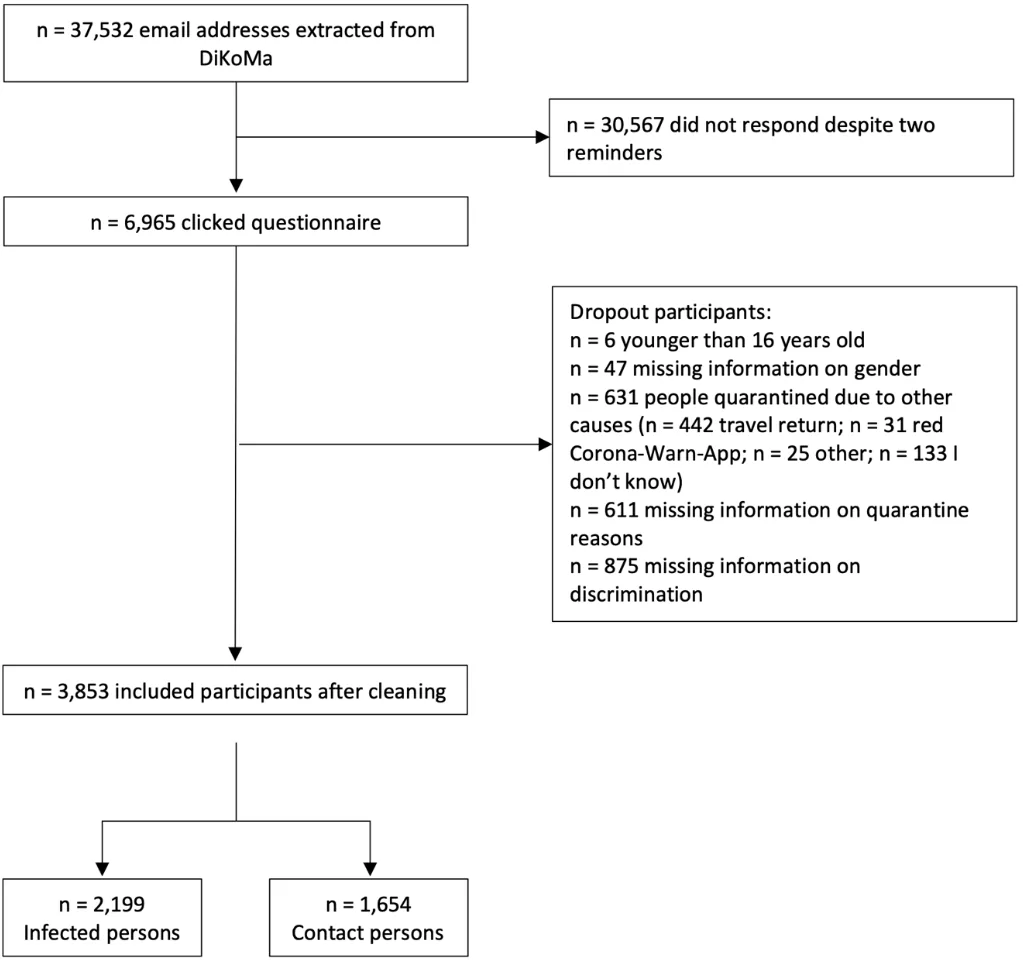
Flowchart of participant inclusion and final analytical sample

**Figure 2:**
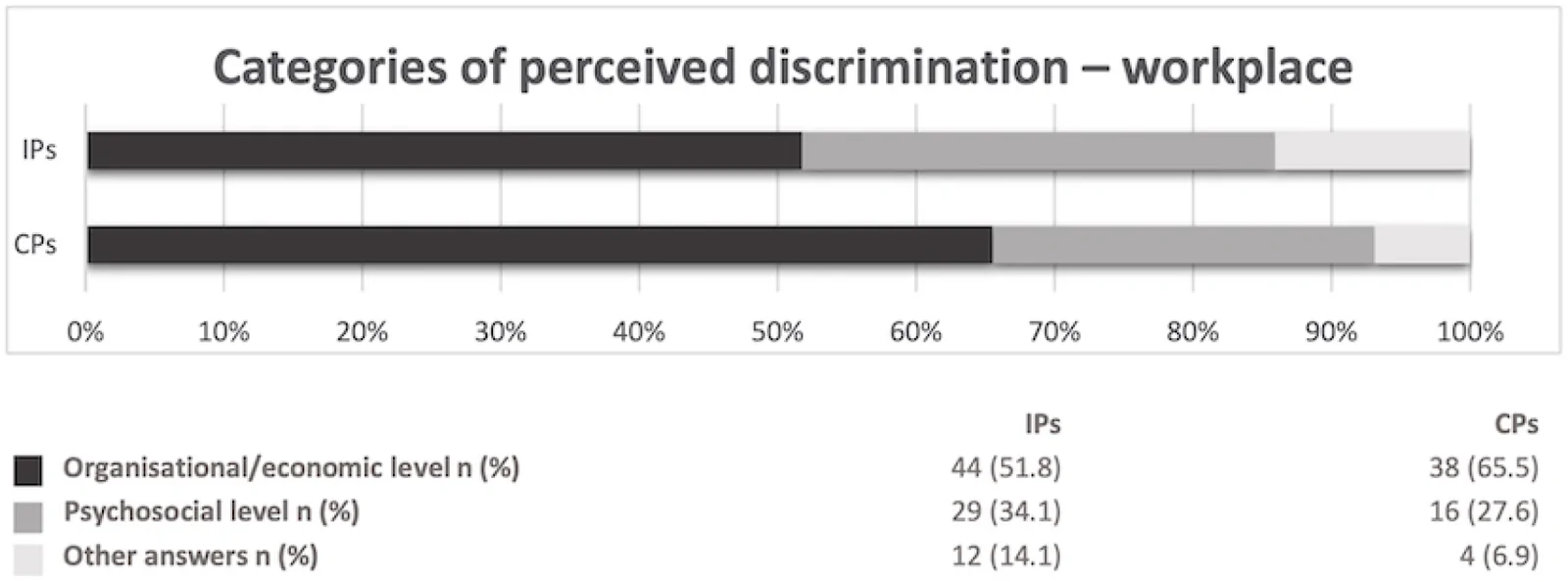
Distribution of perceived discrimination categories in the workplace among infected persons (IPs) and contact persons (CPs)

**Figure 3:**
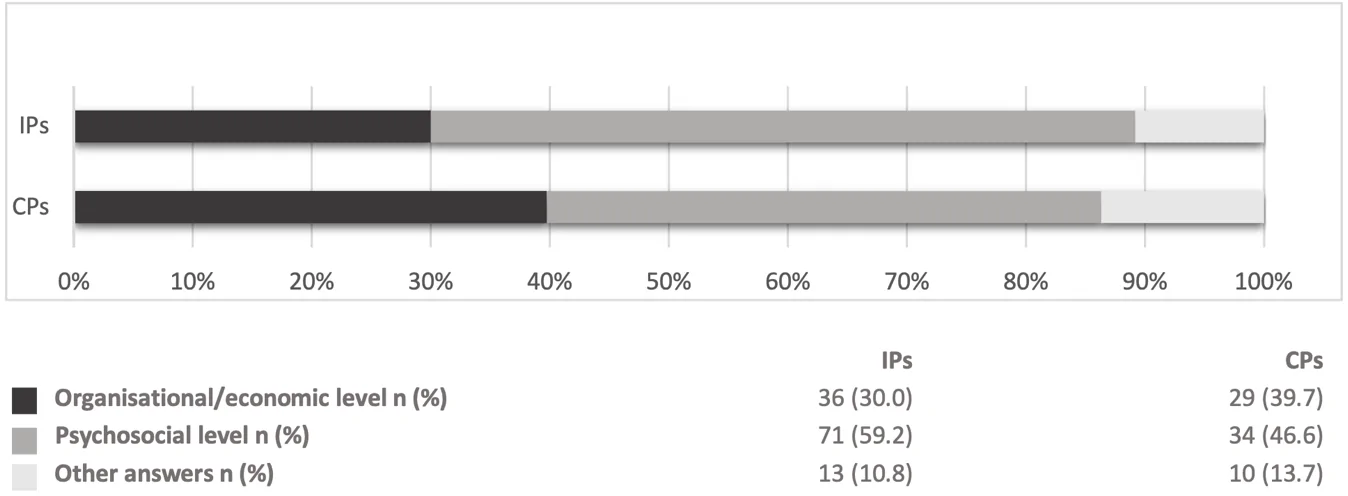
Distribution of perceived discrimination categories in private life among infected persons (IPs) and contact persons (CPs)
